# Diphtheria and Croup

**Published:** 1892-12

**Authors:** 


### DIPHTHERIA AND CROUP.

One of the most conclusive clinical arguments of their identity is afforded by the fact that in some of the large European hospitals cases of croup and diphtheria are placed side by side in the same ward, and the cases of croup do not become infected; while it is not rare for diphtheria to develop in a family in which an apparent case of croup has been present. Fraenkel held autopsies in four cases, clinically typical instances of croup, and perfectly so demonstrated by autopsy. Examination of the membrane present in each case disclosed the presence of the bacillus of diphtheria described by Klebs and Loeffler, the identity of which was assured by its morphologic appearances, by its behavior in culture, and by its pathogenicity to animals.—*Med. News.*

